# Intra-Articular Osteotomy for Distal Humerus Malunion

**DOI:** 10.1155/2009/631306

**Published:** 2009-11-01

**Authors:** René K. Marti, Job Doornberg

**Affiliations:** ^1^Department of Orthopaedic Surgery, Orthotrauma Research Center Amsterdam, Academic Medical Center Amsterdam, University of Amsterdam, Room L107, Meibergdreef 9, 1100 DD Amsterdam, The Netherlands; ^2^Orthopaedic Residency Program, Orthotrauma Research Center Amsterdam, University of Amsterdam, G4-Noord, Kamer 249, Meibergdreef 9, 1100 DD Amsterdam, The Netherlands

## Abstract

Intra-articular osteotomy is considered in the rare case of malunion after a fracture of the distal humerus to restore humeral alignment and gain a functional arc of elbow motion. Traumatic and iatrogenic disruption of the limited blood flow to the distal end of the humerus resulting in avascular necrosis of capitellum or trochlea is a major pitfall of the this technically challenging procedure. Two cases are presented which illustrate the potential problems of intra-articular osteotomy for malunion of the distal humerus.

## 1. Introduction

Distal humerus fractures are uncommon, accounting for only 2 percent of all fractures [1]. Open reduction and internal fixation to restore articular anatomy and allow early motion to prevent stiffness [2] is the preferred treatment [3, 4]. Despite rapid improvements in operative management, fractures of the distal end of the humerus are still among the most difficult fractures to treat. Complex articular anatomy with little cancellous bone support makes operative fixation challenging [1]. Although challenging, in young active patients early fixation is recommended, despite the complexity of the fracture. An unsatisfactory outcome is common for this patient population when minimal treatment is used to treat the initial fracture [5, 6].

In the rare case of malunion, intra-articular or supracondylar osteotomy to restore humeral alignment and provide a more useful arc of motion is legitimate. In the case of an extra-articular osteotomy to correct flexion or extension alignment, treatment can be indicated despite the presence of posttraumatic arthrosis of an incongruent elbow joint. However, intra-articular osteotomy treatment is only considered when the patient is seen early and secondary post-traumatic arthritis is at an early stage [1, 6].

Intra-articular corrective osteotomy for malunited fractures of the distal humerus is technically demanding and the literature on this subject scarce [6–9]. This manuscript documents surgical technique and reports on a case of intra-articular osteotomy for distal humerus malunion.

## 2. Case Report

**Figure 1:**
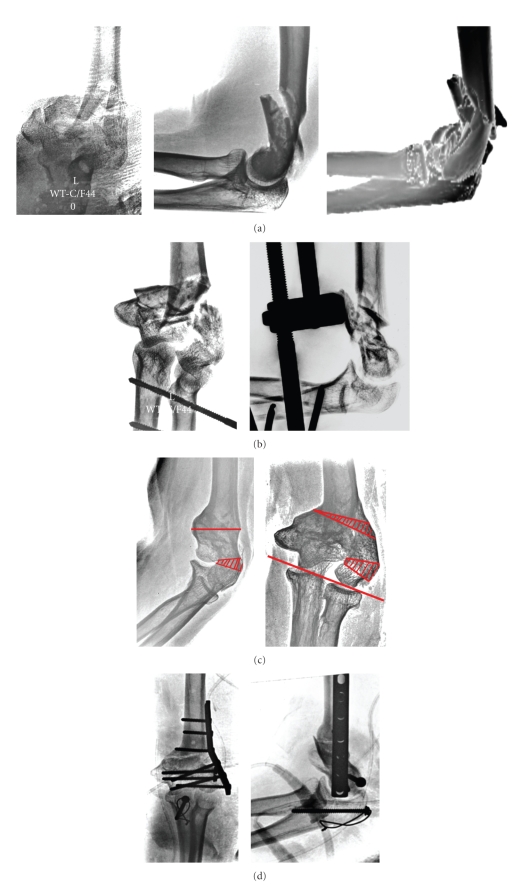

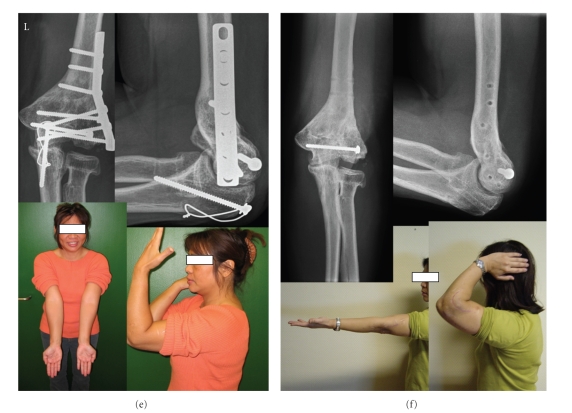
(a) Our patient was a 48-year-old dentist who was seen 10 months after a high energy fall that resulted in a complex intra-articular fracture of her left nondominant distal humerus. (b) Initial treatment performed at an outside institution consisted of static external fixation with the arm in 90° flexion and the forearm in neutral rotation. (c) The external fixator was removed after 8 weeks and follow-up the radiographs revealed distal humerus intra-articular malunion. (d) An oscillating saw was used to create a trapezoid wedge to correct overlength varus of the lateral column and flexion of the capitellum in order to realign capitellum and trochlea. Extreme caution was taken no to disrupt blood supply of the capitellum. (e) The patient had loss of anterior translation of the distal end of the humerus resulting in loss of flexion of 35 degrees, with 10 degrees of hyperextension of the left elbow. (f) Patient opted for removal of hardware and extra-articular excavation of the prominent ventral distal humerus creating a new fossa coronoidea in order to gain flexion.

Our patient was a 48-year-old dentist who was seen 10 months after a high energy fall that resulted in a complex type C3 intra-articular fracture of her left nondominant distal humerus (Figure 1(a)). Initial treatment performed at an outside institution consisted of static external fixation with the arm in 90° flexion and the forearm in neutral rotation (Figure 1(b)). The external fixator was removed after 8 weeks and the follow-up radiographs revealed delayed union which resulted in intra-articular malunion of the distal humerus. The patient was referred to the senior author (RKM) with substantial overlength of the capitellum resulting in 25 degrees cubitus varus deformity. Flexion extension arc was 95 degrees with a 35-degree flexion contracture. Her forearm rotation was normal. She did not have pain and did not opt for surgery at this point. Four months later, she reported substantial pain and lost all her active motion. The patient opted for surgery mainly to regain extension in order to return to work. Neurological examination was unremarkable. She had no signs of ulnar neuropathy.

She underwent corrective a transverse osteotomy 18 months after her initial injury. A midline posterior incision, with medial and lateral skin flaps elevated, was used. The olecranon osteotomy provided a complete overview of the dorsal distal humerus, including the radiohumeral joint and the ulnar nerve. An oscillating saw was used to create a trapezoid wedge to correct overlength varus of the lateral column and flexion of the capitellum in order to realign capitellum and trochlea. Extreme caution was taken no to disrupt the anterior blood supply of the capitellum. A second supracondylar wedge was resected to correct cubitus varus and 20 degrees of internal rotation. The capitellum radii was fixed to the ulnar condyle using an isolated lag screw (Figure 1(c)). Iliac crest bone graft was used to improve bone healing and fixation was achieved with a lateral 7hour-LCDC plate and screws. The olecranon osteotomy was fixed with screw-tension band technique (Figure 1(d)). Intraoperative ulnohumeral motion was 90 degrees of flexion with full extension after debridement of the olecranon fossa. The elbow was placed in a cast in full extension for 24 hours.

She had loss of anterior translation of the distal end of the humerus resulting in 90 degrees of flexion and 5 degrees of hyperextension of the left elbow (Figure 1(e)). The patient was satisfied initially with her stable function elbow and 95 degrees of ulnohumeral motion. However, 18 months postoperatively she decided to have removal of hardware and an extra-articular excavation of the prominent ventral distal humerus creating a new fossa coronoidea in order to gain flexion. Her final flexion improved to 110 degrees with 5 degrees of hyperextension (Figure 1(f)). She had a functional outcome, quantified as 95 points by the American Shoulder and Elbow Surgeons Elbow Evaluation Instrument and the Mayo Elbow Performance Index. Continuous passive motion was initiated 24 hours after surgery and therapy was started.

## 3. Discussion

To the best of our knowledge, this is the first report to specifically address intra-articular osteotomy to treat distal humerus malunion. However, small series have been published as part of surgical technique and clinical outcome papers on intra- and extra-articualar malunion [5]. McKee and Jupiter reported on two malunited fractures of the distal humerus that underwent intra-articular osteotomy in a series of distal humerus malunion and nonunion with good results [6].

The cornerstone of treatment of fractures of the distal humerus is operative reduction and internal fixation [6–13]. Pessimism regarding these fractures is not justified with contemporary methods of internal fixation [10]. However, routine in treatment does not exist due to the rarity of these fractures. In addition, the complex articular surface and meager amount of cancellous bone still makes operative treatment a substantial challenge.

Nonunion and malunion of distal humerus fractures treated operatively is rare. Only 2% of all fractures involve the distal humerus, and 2% of these result in malunion. Intra-articular osteotomy for malunion to achieve a functional arc of motion and to prevent subsequent posttraumatic arthritis in the elbow joint is rarely indicated in the young demanding patient. Therefore, experience with treatment of intra-articular malunited fractures is very limited and reports seldom appear in literature [6–9]. We feel that total elbow arthroplasty for malunion of the distal humerus is a treatment option reserved for low-demand elderly patients [12].

If the patient present at an early stage, an intra-articular osteotomy is considered if original fracture lines can be osteotomized and posttraumatic arthrosis is limited. Extra-articular osteotomy is indicated when intra-articular congruency of the elbow joint cannot be restored and secondary posttraumatic arthrosis is too advanced [1, 6]. Preoperative planning is paramount to assess if it is technically feasible to restore ulnohumeral congruency (Figure 1(c)).

Stability of the elbow joint and a functional arc of motion were achieved in this case. After intra-articular osteotomy, the patient had only 90 degrees of flexion due to loss of anterior translation of the distal humerus [13]. Initial loss of flexion was accepted by patient and surgeon to avoid devascularization of the distal end of the humerus. After avoiding too much dissection during the index procedure in case one, the patient opted for a successful subsequent procedure to gain flexion. Satisfactory functional outcome was quantified by the Mayo Elbow Performance Index and American Shoulder and Elbow Surgeons Evaluation Instrument.

Surgical reconstruction of the intra-articular malunited distal humerus is technically challenging but can improve function in the young active adult by restoring intrinsic anatomy of the elbow.
